# Prognostic value of circulating endothelial cells in metastatic colorectal cancer

**DOI:** 10.18632/oncotarget.16397

**Published:** 2017-03-21

**Authors:** Nuh N Rahbari, Sebastian Schölch, Ulrich Bork, Christoph Kahlert, Martin Schneider, Mohammad Rahbari, Markus W Büchler, Jürgen Weitz, Christoph Reissfelder

**Affiliations:** ^1^ Department of Gastrointestinal, Thoracic and Vascular Surgery, Technische Universität Dresden, Dresden, Germany; ^2^ Department of General, Visceral and Transplant Surgery, University of Heidelberg, Heidelberg, Germany

**Keywords:** circulating endothelial cells, CEC, circulating tumor cells, CTC, colorectal cancer

## Abstract

**BACKGROUND:**

There is urgent need for improved staging in patients with metastatic colorectal cancer (mCRC). In this study, we evaluated the prognostic value of circulating endothelial cells (CEC) in comparison with circulating tumor cells (CTC) in patients with mCRC amenable for potentially curative surgery.

**METHODS:**

A total of 140 patients were enrolled prospectively. CTC and CEC were measured with the CellSearch System (Veridex, NJ, USA). Cut-off values were determined using ROC analyses. Prognostic factors were identified by Cox proportional hazards models.

**RESULTS:**

ROC analyses revealed ≥ 21 CEC as cut-off levels for detection, which was present in 68 (49%). CEC detection was associated with female gender (p = 0.03) only, whereas CTC detection was associated with presence of the primary tumor (p = 0.007), metastasis size (p < 0.001), bilobar liver metastases (p = 0.02), CEA (p < 0.001) and CA 19-9 levels (p < 0.001). On multivariate analysis only CEC detection (HR 1.81; p = 0.03) and preoperative CA19-9 levels (HR 2.28, p = 0.005) were revealed as independent predictors of poor survival.

**CONCLUSIONS:**

CEC are of stronger prognostic value than CTC. Further studies are required to validate these results and to evaluate CEC as predictive biomarker for systemic therapy alone as well as in combination with other markers such as CA19-9.

## INTRODUCTION

Solid tumors are critically dependent on angiogenesis to exceed a size of 2-3 mm^3^ [1, 2]. In addition to vascular injury, which regularly occurs in all solid tumors and may be a result of overshooting angiogenesis, all modes of tumor angiogenesis induce shedding of cells of endothelial origin into the circulation. These circulating endothelial cells (CEC) may therefore be used as surrogate marker for the tumor's angiogenic activity as well as the degree of vascular injury. In addition, endothelial progenitor cells (EPC) from the bone marrow are recruited to tumors by pro-angiogenic cytokines [3].

Numerous different phenotypes have been used to define, quantify and isolate CEC (reviewed in reference [4]). As the methods of CEC detection and the patient populations studied vary vastly [4], comparisons between CEC studies have been difficult.

CRC metastases are highly angiogenic, which may explain the clinical effectiveness of antiangiogenic therapies in this disease [5]. In the past, CEC numbers have been shown to be predictive of the clinical activity of antiangiogenic agents [6–9]. However, the prognostic value of CEC in CRC patients with metastatic disease who are amenable to curative therapy has never been investigated.

It was therefore the aim of the present study to investigate the prognostic value of CEC in patients with CRC liver metastases who underwent potentially curative therapy using the semi-automated and highly reproducible CellSearch System for CEC detection [8,10–12]. As CTC are well-established prognostic markers in primary and metastatic CRC [13–22], we aimed to evaluate the prognostic value of CEC in a ‘head-to-head’ comparison with CTC in the same cohort of patients with metastatic CRC.

## RESULTS

### Study population

A total of 140 patients were enrolled between September 2009 and August 2012 (Supplementary Table 1). There were 80 (57.1%) men and the median age was 62 (28 – 82) years. The primary tumor was located in the colon in 96 (68.6%) patients and was node-positive in 92 (65.7%). 96 (68.6%) patients had previously undergone resection of the primary tumor. Bilobar metastases were present in 39 (27.9%) patients and extrahepatic disease in 15 (10.8%) patients. 55 (39.3%) patients had received neoadjuvant therapy. Data on the *KRAS* mutational status was available for 81 patients of whom 47 (58%) had *KRAS* wild-type tumors. None of the patients required vascular resection/reconstruction.

### Preoperative detection of CEC and CTC and association with clinicopathologic variables

**Figure 1:**
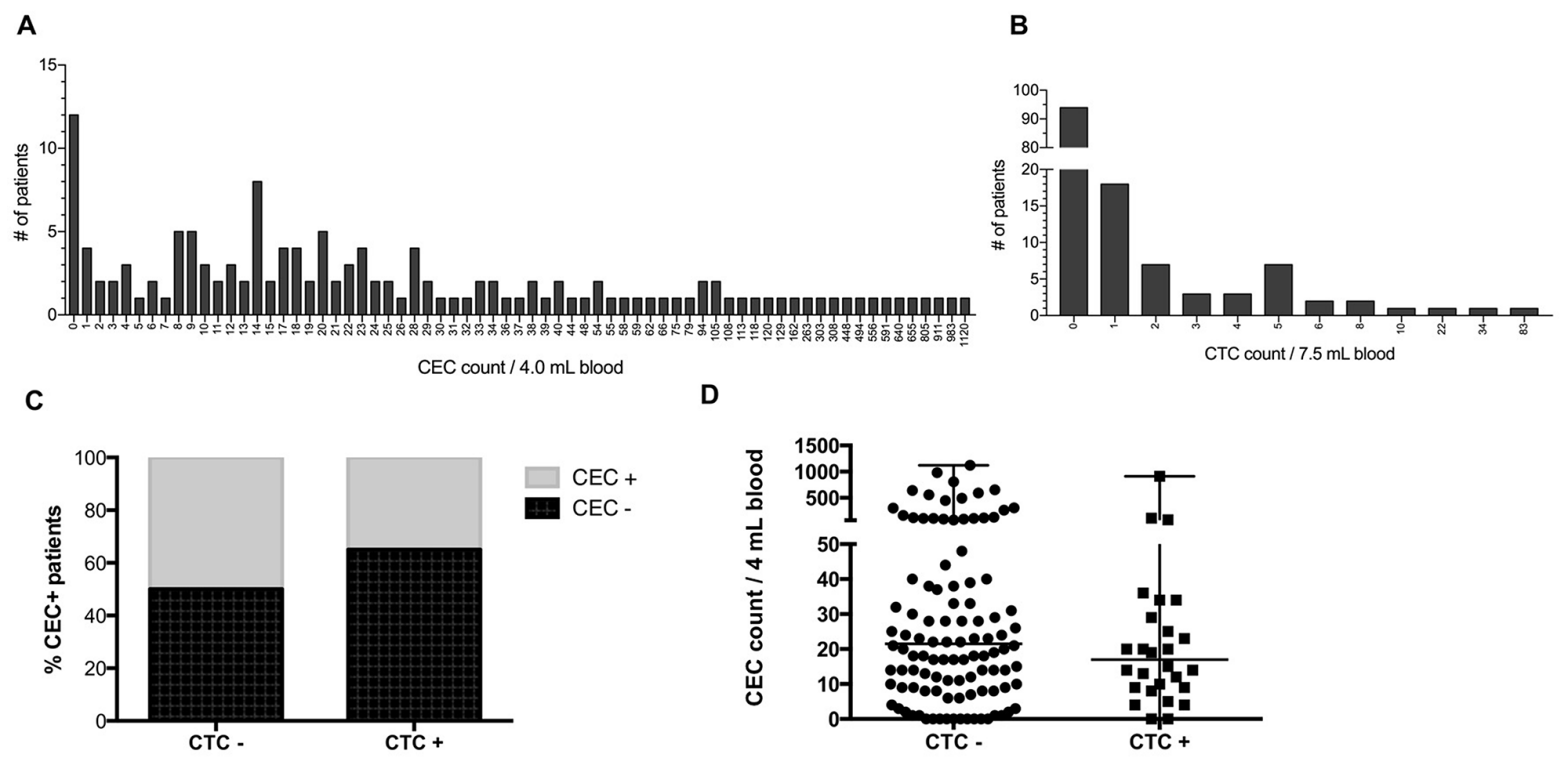
Detection of CEC and CTC in patients with colorectal liver metastases **(A)** Histogram of CEC counts per 4 mL blood. **(B)** Histogram of CTC counts per 7.5 mL blood. **(C)** CTC-dependent CEC detection. **(D)** CTC-dependent CEC count.

ROC analyses revealed a cut-off of ≥ 21 for detection of CEC (AUC 0.672; 95% CI 0.55 – 0.79) with a true positive rate of 64.3% and a true negative rate of 45.5%, respectively (Supplementary Figure 1). Based on previous reports using the CellSearch technology a cut-off of ≥ 2 was used for CTC detection [15, 17, 19, 23]. Using these cut-off values 66 (47.1%) patients were positive for CEC and 28 (20%) patients were positive for CTC. CEC numbers ranged from 0 to 1,120 with a median of 20 and a mean of 82.4 CEC (Figure 1A). Only 12 (8.6%) patients had no detectable CEC. CTC values varied between 0 and 83 with a median and mean value of 0 and 1.9, respectively (Figure 1B). Neither CEC detection rates (p = 0.16), nor CEC counts (p = 0.33) differed significantly in CTC-positive as compared to CTC-negative patients (Figure 1C and 1D). Moreover, CEC detection rate was not significantly different in patients with and without cardiovascular comorbidities (56.2% vs. 45.7%; p = 0.44).

To evaluate whether detection of CEC and CTC reflect the extent of tumor burden or might serve as independent prognostic and predictive biomarkers, we next assessed if detection of CEC and CTC is associated with clinicopathologic variables (Table 1). In line with published data on endothelial progenitor cells (which are included by the markers used for CEC detection by the CellSearch system) there was a higher CEC detection rate (p = 0.03) and CEC count (p = 0.009) in women as compared to men [24, 25]. Also, there was a trend toward a lower CEC detection rate (p = 0.08) and CEC count (p = 0.09) in patients who received neoadjuvant chemotherapy together with bevacizumab. CEC detection was not significantly different in patients with and without sinusoidal obstruction syndrome after previous chemotherapy (p = 0.27). There was neither a significant association of a staged vs. simultaneous resection with CEC detection (p = 0.14) nor with CTC detection (p = 0.5). The *KRAS* mutational status was not associated with detection of CEC (p = 0.39) or CTC (p = 0.59). As reported previously [26], CTC detection in this cohort of metastatic CRC patients was associated with presence of the primary tumor (p = 0.007), metastasis size (p < 0.001), bilobar liver metastases (p = 0.02), CEA (p < 0.001) and CA 19-9 levels (p < 0.001). Similarly, CTC counts were higher in patients without resection of the primary tumor (p = 0.01), metastasis size (p < 0.001), bilobar distribution of metastases, CEA (p < 0.01) and CA 19-9 levels (p < 0.001). Furthermore, CTC detection rate (p < 0.001) and (p = 0.002) count was associated with the patients’ MSKCC risk score.

**Table 1:** Univariate analyses of clinicopathologic factors associated with detection rate and count of CEC and CTC

	CEC +				CTC +			
	N (%)	p	Mean (median;range)	p	N (%)	p	Mean (median;range)	p
**Gender**								
Male	31 (38.8)	0.03	77.9 (17; 0 – 1120)	0.009	11 (13.8)	0.05	0.7 (0; 0 – 8)	0.06
Female	35 (58.3)		88.3 (24; 0 – 911)		17 (28.3)		3.4 (0; 0 – 83)	
**Age [years]**								
< 65	38 (44.2)	0.39	68 (18.5; 0 – 983)	0.28	17 (19.8)	0.97	2.1 (0; 0 – 83)	0.82
≥ 65	28 (51.6)		105.3 (23; 0 – 1120)		11 (20.4)		1.5 (0; 0 – 34)	
**Disease-free interval [months]**								
< 12	44 (47.8)	0.86	94.2 (20; 0 – 1120)	0.25	21 (22.8)	0.28	1.9 (0; 0 – 83)	0.47
≥ 12	22 (45.8)		59.8 (18.5; 0 – 655)		7 (14.6)		1.8 (0; 0 – 34)	
**Node-positive primary**								
Yes	47 (51.1)	0.22	92.0 (22.5; 0 – 983)	0.19	20 (21.7)	0.51	2.0 (0; 0 – 83)	0.96
No	19 (39.6)		63.9 (17.5; 0 – 1120)		8 (16.7)		1.6 (0; 0 – 34)	
**Site of primary tumor**								
Colon	42 (43.8)	0.28	61.3 (19.5; 0 – 1120)	0.12	23 (23.9)	0.11	2.1 (0; 0 – 83)	0.08
Rectum	24 (54.6)		128.4 (23; 0 – 805)		5 (11.4)		1.3 (0; 0 – 34)	
***KRAS*** **mutation status^1^**								
Wild-type *KRAS*	19 (40.4)	0.39	68.1 (18; 0 – 1258)	0.47	10 (21.3)	0.59	1.4 (0; 0 – 22)	0.94
Mutant *KRAS*	17 (50)		126 (20.5; 0 – 983)		9 (26.7)		4.4 (0; 0 – 83)	
**Previous resection of primary**								
Yes	48 (50.0)	0.36	60.7 (21.5; 0 – 655)	0.57	13 (13.5)	0.007	1.0 (0; 0 – 34)	0.01
No	18 (40.9)		129.8 (19; 0 – 1120)		15 (34.1)		3.8 (0; 0 – 83)	
**Number of metastases**								
1	25 (43.9)	0.61	79.6 (18; 0 -1120)	0.16	10 (17.5)	0.67	0.8 (0; 0 – 8)	0.62
> 1	41 (49.4)		84.3 (21; 0 -983)		18 (21.7)		2.6 (0; 0 – 83)	
**Size of largest metastasis [cm]**								
< 5	47 (45.6)	0.57	64.0 (20; 0 – 1120)	0.13	10 (9.7)	< 0.001	0.6 (0; 0 – 8)	< 0.001
≥ 5	19 (51.4)		133.5 (24; 0 – 983)		18 (48.6)		5.5 (1; 0 – 83)	
**Distribution of metastases**								
Unilobar	50 (49.5)	0.45	82.9 (21; 0 – 1120)	0.93	15 (14.9)	0.02	0.7 (0; 0 – 8)	0.03
Bilobar	16 (41.0)		81.2 (19; 0 – 911)		13 (33.3)		4.9 (0; 0 – 83)	
**Extrahepatic disease**								
Yes	9 (60.0)	0.41	64.9 (25; 0 – 448)	0.61	1 (6.7)	0.3	2.1 (0; 0 – 83)	0.07
No	57 (45.9)		85.1 (20; 0 – 1120)		27 (21.8)		0.2 (0; 0 – 2)	
**CEA level [μg/l]**								
< 2.5	18 (46.2)	0.98	93.6 (18; 0 – 1120)	0.92	1 (2.6)	<0.001	0.2 (0; 0 – 2)	0.01
≥ 2.5	47 (47.9)		80.0 (20; 0 – 983)		26 (26.5)		2.6 (0; 0 – 83)	
**CA 19-9 level [μg/l]**								
< 37	42 (45.2)	0.47	88.3 (18; 0 – 1120)	0.65	10 (10.8)	<0.001	0.8 (0; 0 – 22)	<0.001
≥ 37	23 (52.3)		74.5 (23; 0 – 911)		17 (38.6)		4.3 (1; 0 – 83)	
**MSKCC risk score**								
0	2 (40.0)	0.08	24.2 (21; 0 – 54)	0.06	1 (20.0)	<0.001	0.8 (0; 0 – 3)	0.002
1	7 (28.0)		89.4 (11; 0 – 1120)		1 (4.0)		0.4 (0; 0 -8)	
2	27 (50.0)		64.3 (21; 0 – 655)		6 (11.1)		0.6 (0; 0 – 8)	
3	23 (60.5)		77.9 (31.5; 0 - 805)		8 (21.1)		1.9 (0; 0 – 34)	
4	4 (28.6)		87.8 (14.5; 0 – 983)		11 (78.6)		10.3 (3.5; 0 – 83)	
5	3 (75.0)		379.5 (298; 0 – 911)		1 (25.0)		0.8 (0; 0 – 3)	
**Neoadjuvant chemotherapy**								
Yes	28 (42.4)	0.29	99.1 (17; 0 – 983)	0.48	10 (15.2)	0.18	0.7 (0; 0 – 10)	0.12
No	38 (51.3)		67.5 (23; 0 – 1120)		18 (24.3)		2.9 (0; 0 – 83)	
**Neoadjuvant Bevacizumab**								
Yes	13 (35.1)	0.08	79.2 (15; 0 – 805)	0.09	6 (16.2)	0.52	0.8 (0; 0 – 10)	0.11
No	53 (51.5)		83.6 (23; 0 – 1120)		22 (21.4)		2.3 (0; 0 – 83)	
**Neoadjuvant Cetuximab**								
Yes	6 (42.9)	0.69	172.5 (15.5; 0 – 983)	0.71	2 (14.3)	0.56	0.6 (0; 0 – 3)	0.89
No	60 (48.4)		73.4 (20.5; 0 – 1120)		26 (20.9)		2.1 (0; 0 – 83)	

Data are presented as n (%) or mean (median; range); CEA, Carcinoembryonic antigen; MSKCC, Memorial Sloan-Kettering Cancer Center

^1^Data missing for 59 patients.

### Prognostic value of CEC and CTC in patients with resectable colorectal liver metastases

**Figure 2:**
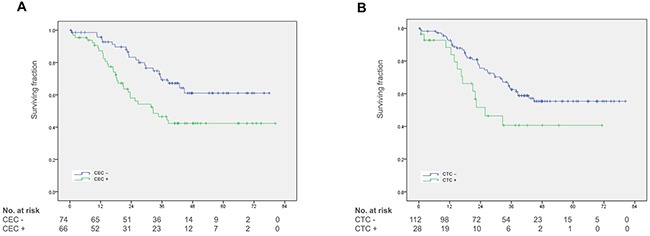
Kaplan-Meier plots for overall survival **(A)** Overall survival in patients with and without CEC detection. **(B)** Overall survival in patients with and without CTC detection.

The median duration of follow-up was 32 (0.5 – 80) months for the entire study cohort and 41 (0.4 – 80) months for survivors. Some 56 (40%) patients died during the follow-up period. On univariate analyses a significant association with overall survival was found for detection of ≥ 21 CEC (45.2 vs. 58.2 months; p = 0.005) and detection of ≥ 2 CTC (39.2 vs. 55.2 months; p = 0.03) (Figure 2A and 2B). The association of CEC with survival was confirmed using various cut-off levels such as the 30^th^ percentile of CEC counts (48.9 vs. 60.4 months; p = 0.03), the median CEC count (44.7 vs. 60.1 months; p = 0.001) and the 70^th^ percentile of CEC counts (44.1 vs. 55.1 months; p = 0.02). However, there was no or only moderate association of CTC detection with survival using ≥ 1 CTC (46.1 vs. 53.7 months; p = 0.06) and ≥ 3 CTC (33.4 vs. 54.3 months; p = 0.13) as cut-off values (Supplementary Table 2). To further explore the impact of neoadjuvant therapy with and without VEGF-targeted therapy on the prognostic value of CEC in metastatic CRC patients we performed further subgroup survival analyses. These analyses confirmed the prognostic value of CEC in the subset of patients without neoadjuvant chemotherapy (43.6 vs. 59.7 months; p = 0.04) as well as patients without neoadjuvant therapy with bevacizumab (45.8 vs. 61.1 months; p = 0.007). In addition, univariate analyses revealed multiple metastases (46.3 vs. 59.2 months; p = 0.04), CEA levels ≥ 2.5 μg/L (45.2 vs. 63.0 months; p = 0.01), CA 19-9 levels ≥ 37 μg/L (33.3 vs 59.3 months; p < 0.001) and a MSKCC risk score > 2 (41.4 vs. 59.1 months, p = 0.002) to be associated with a poor prognosis (Table 2).

**Table 2:** Univariate analyses of overall survival

	3-year survival [%]	Mean survival[months]	P
**Gender**			
Male	58.7	52.1 (45.4 – 58.8)	0.78
Female	58.5	52.4 (43.9 – 61.0)	
**Age [years]**			
< 65	56.6	51.1 (44.3 – 57.9)	0.72
≥ 65	61.3	53.6 (45.0 – 62.2)	
**Disease-free interval [months]**			
< 12	53.6	50.6 (43.7 – 57.4)	0.23
≥ 12	67.4	54.9 (46.1 – 63.7)	
**Node-positive primary**			
Yes	58.2	52.7 (43.6 – 61.7)	0.78
No	58.7	51.9 (45.2 – 58.6)	
**Site of primary tumor**			
Colon	59.1	52.8 (46.0 – 59.6)	0.98
Rectum	55.4	49.1 (41.0 – 57.2)	
**Previous resection of primary**			
Yes	62.2	54.7 (48.1 – 61.2)	0.32
No	51	46.3 (37.5 – 55.2)	
**Number of metastases**			
1	71.4	59.2 (51.0 – 67.3)	0.04
> 1	49.4	46.3 (39.6 – 52.9)	
**Size of largest metastasis [cm]**			
< 5	61.9	54.5 (48.4 – 60.6)	0.11
≥ 5	45.4	46.4 (35.1 – 57.8)	
**Distribution of metastases**			
Unilobar	62.1	54.6 (48.3 – 60.9)	0.25
Bilobar	48.1	45.9 (36.6 – 55.8)	
**Extrahepatic disease**			
Yes	53.3	34.1 (24.5 – 43.6)	0.69
No	71	52.9 (47.2 – 58.6)	
**CEA level [μg/l]**			
< 2.5	75.7	63.0 (54.3 – 71.7)	0.01
≥ 2.5	48.3	45.2 (39.1 – 51.3)	
**CA 19-9 level [μg/l]**			
< 37	69.3	59.3 (53.0 – 65.5)	< 0.001
≥ 37	33.3	33.3 (26.1 – 40.6)	
**MSKCC risk score**			
≤ 2	70.3	59.1 (52.4 – 65.7)	0.002
> 2	40	41.4 (33.3 – 49.4)	
**CEC**			
< 21	69.3	58.2 (51.4 – 64.9)	0.005
≥ 21	44.5	45.2 (47.4 – 58.3)	
**CTC**			
< 2	62.6	55.2 (49.4 – 61.1)	0.03
≥ 2	40.6	39.2 (27.7 – 50.8)	

Data are presented as n (%) or mean (median; range); Analyses were performed using the log-rank test.

CEA, Carcinoembryonic antigen; MSKCC, Memorial Sloan-Kettering Cancer Center; CEC, Circulating endothelial cells; CTC, Circulating tumor cells.

We next constructed a multivariate Cox proportional hazards model to assess the independent prognostic relevance of CEC and CTC in the context of the identified clinicopathologic factors with prognostic value in this patient cohort. This ‘head-to-head’ comparison confirmed CEC as independent prognostic biomarker (HR 1.81, 95% CI 1.05 – 3.14; p = 0.03), whereas CTC were of no prognostic value (HR 1.34, 95% CI 0.65 – 2.77; p = 0.43). However, this model revealed elevated CA 19-9 levels as strongest prognostic biomarker (HR 2.28, 95% CI 1.28 - 4.06; p = 0.005) (Table 3).

**Table 3:** Multivariate analysis of factors associated with overall survival

Variable	Comparison	Hazard ratio	95% CI	*p* Value
Number of metastases	1 vs. ≥ 2	1.52	0.81 – 2.89	0.19
MSKCC clinical risk score	> 2 vs. ≤ 2	1.37	0.72 – 2.62	0.34
CA 19-9 [μg/l]	< 23 vs. ≥ 23	2.28	1.28 – 4.06	0.005
CTC	≥ 2 vs. < 2	1.34	0.65 – 2.77	0.43
CEC	≥ 21 vs. <21	1.81	1.05 – 3.14	0.03

Metastasis size and CEA were not considered in the model as they are included in the MSKCC risk score.

Memorial Sloan-Kettering Cancer Center; CEC, Circulating endothelial cells; CTC, Circulating tumor cells.

## DISCUSSION

We here show that high CEC numbers are an independent prognostic factor of poor survival that outperform CTC as prognostic biomarker in metastatic CRC after adjustment for other clinical variables. In contrast to CTC, CEC are independent of other tumor-related clinical variables assessed in the present study. The potential of CEC to provide biological information in addition to commonly used factors to assess the extent of disease underlines its potential as independent biomarker that may improve staging and precision medicine approaches.

Two studies on patients with non-small cell lung cancer and breast cancer have already demonstrated that CEC detection with the CellSearch system is not associated with other clinicopathologic variables commonly used for staging of patients, even though CEC in these studies were of significant prognostic value [11, 27]. Our results are clearly in line with these data. The currently available data about CEC in CRC as well as other tumor entities is not only very limited but also highly heterogeneous due to differences in patient cohorts and CEC detection assays [28–34]. Most studies involving CEC in CRC used flow cytometry protocols with varying sets of surface markers [4,6,7,28–32], resulting in data which is difficult to compare. To date, only two publications present CEC numbers in CRC patients obtained in a standardized manner using the CellSearch System [8, 9]. Interestingly, the studies had contradictory results, Matsusaka et al. demonstrated a prognostic value of CEC only for patients who received FOLFOX together with bevacizumab [8], whereas Simkens et al. presented no predictive value of CEC in patients treated with chemotherapy and bevacizumab [9]. There are several reasons that might explain the differences between our data and the results of these studies. First, both studies enrolled patients with palliative therapy. This is fundamentally different from our study which is the first to investigate the prognostic significance of CEC in metastatic CRC patients amenable to curative surgery. Second, in both studies patients were treated with systemic chemotherapy, whereas about half of our patients received no preoperative chemotherapy and chemotherapy protocols in the remaining half included irinotecan and EGFR-targeting agents in a significant proportion of patients. Finally, Matsusaka et al. used a cut-off level of 65 CEC / 4 mL of blood, whereas Simkens et al. did not use a cut-off value. We used ROC analyses to determine a cut-off level of 21 CEC / 4 mL blood for our study. Collectively, these differences in study designs render cross-comparisons between our study and the published studies difficult and may explain the observed differences.

CEC must be distinguished from endothelial progenitor cells (EPC), which share many surface markers with CEC, but are a distinct cell population of different origin and function: CEC are thought to be shed from the vasculature and are incapable of colony formation, whereas EPC are bone marrow-derived, immature cells [35]. While CEC may reflect damage of peripheral vessels, there is evidence that EPC contribute to neovascularization [36, 37]; although contradictory data has been published as well [38]. The phenotype of EPC involves endothelial markers, but by definition also includes stem cell markers such as CD133 or CD34 [35]. The phenotype detected by the CellSearch System therefore encompasses both CEC and EPC as it uses the pan-endothelial marker CD146 and does not exclude stem cells [39].

The biological role of CEC remains controversial. There is some consensus that mature CEC may reflect the level of vascular injury whereas circulating endothelial progenitors may reflect the level of vascular repair and neovascularization [32,35–37,40]. CEC originating from damaged tumor vasculature may be responsible for our finding of a therapy-induced reduction of CEC by bevacizumab as the tumor vasculature is also reduced and normalized during VEGF-targeted therapy [41, 42]. Conversely, highly vascularized tumors have better nutrient and oxygen supply as well as better access to the vascular system. Such tumors will shed more CEC and have a worse prognosis, explaining the prognostic value of CEC. However, this study did not investigate mechanistic processes and therefore does not allow conclusions about the actual role of CEC in disease progression of patients with metastatic CRC.

Elevated preoperative CA19-9 levels were found to be the strongest prognostic marker in our survival analyses. This is in agreement with multiple previous studies on patients with primary and metastatic CRC [43–52]. As CA19-9 levels did not correlate with CEC detection in our study, both markers may provide complementary information and thus be of potential use as combined biomarkers in future management of patients with metastatic CRC.

In conclusion, CEC numbers as measured by the CellSearch System are an independent prognostic factor in metastatic colorectal cancer and as such may be superior to CTC. CEC may thus serve as valuable addendum to the currently available panel of prognostic markers is mCRC. Future studies are required to elucidate subgroups of CEC with particularly strong biological and thus prognostic relevance. Furthermore, future studies need to evaluate the predictive value of CEC as a single biomarker as well as in combination with other markers such as CA19-9.

## PATIENTS AND METHODS

### Patients

Our management of patients with colorectal liver metastases has been reported previously [14, 53]. Briefly, patients with histologically confirmed colorectal liver metastases who underwent curative surgery at the Department of General Surgery, University Hospital Heidelberg between September 2009 and August 2012 were eligible for inclusion in this prospective study. All procedures were performed as open surgery. Exclusion criteria included emergency surgery, unresectable disease, or a history of any other malignancy within the past 5 years. The study was approved by the independent ethics committee of the University of Heidelberg. All patients provided written informed consent prior to surgery.

### Blood sampling and quantification of circulating endothelial cells and circulating tumor cells

All blood samples were obtained directly prior to surgery. Blood sampling and cell quantification using the CellSearch System have been described in detail before [23]. Briefly, blood samples for CEC and CTC detection were drawn from a central venous catheter into 7.5 mL cell preservative tubes (CellSave Tubes, Veridex, NJ) prior to the first incision. Samples were maintained at room temperature and processed within 96 hours (CTC) and 72 hours (CEC). The analysis via the CellSearch System (CEC and CTC Kits, Veridex, NJ) was conducted in an operator-blinded fashion by specifically trained and Veridex-certified staff. CTC were defined as EpCAM^+^CK^+^DAPI^+^CD45^-^ and CEC as CD146^+^CD105^+^DAPI^+^CD45^-^ events. Each sample was analyzed by two independent operators. Disagreements were resolved by discussion. Results of CTC and CEC detection were expressed as per 7.5 mL blood (CTC) and per 4 mL blood (CEC).

### Statistical analysis

Categorical data were presented as absolute and relative frequencies and compared using the *χ^2^*-test. Continuous data were presented as median and range and compared using the Wilcoxon test. In addition, the arithmetic mean was reported for CEC and CTC counts. The primary endpoint of the present study was overall survival defined as the time interval from the date of operation until death. Receiver operating curve (ROC) analyses were used to determine cutoff values with optimal sensitivity and specificity for the association of CEC and CTC detection with mortality [54]. In line with previous studies cut-off levels for CEA and CA19-9 values were chosen based on the reference ranges provided of our hospital laboratory [43, 44, 51]. Survival curves were constructed according to the Kaplan-Meier method and compared using the log-rank test. Variables with significant associations with survival on univariate analyses were included in a multivariate Cox proportional hazards regression analysis together with CEC and CTC as additional factors. A p-value ≤ 0.05 was considered to indicate a statistically significant difference. All p-values were two-sided. Statistical analyses were done with SPSS^®^ software version 23 (SPSS, Chicago, USA) and JMP program version 7 (SAS Institute Inc., Cary, USA).

## SUPPLEMENTARY MATERIALS FIGURES AND TABLES


