# Supplementary material for: Prognostic value of circulating endothelial cells in metastatic colorectal cancer
Source: Oncotarget. 2017 Mar 21;8(23):37491–501. doi: 10.18632/oncotarget.16397 (PMC5514924; doi:10.18632/oncotarget.16397)
Supplement: Supplementary file 1 [file oncotarget-08-37491-s001.pdf]

Prognostic value of circulating endothelial cells in metastatic colorectal cancer

SUPPLEMENTARY FIGURE AND TABLES

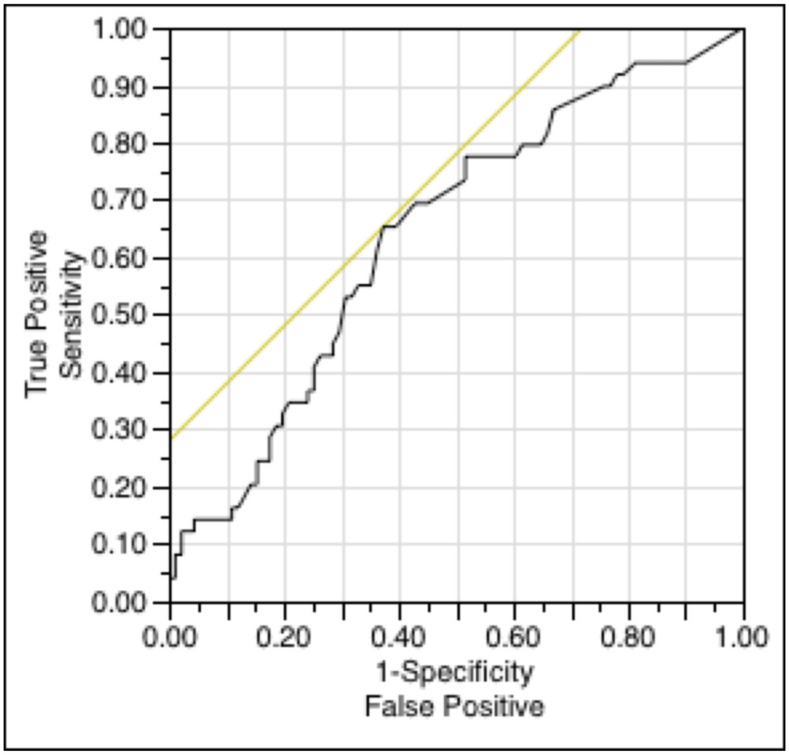

AUC: 0.64; 95% CI (0.54 – 0.73)

Supplementary Figure 1: ROC analysis of CEC detection on overall survival.

Supplementary Table 1: Clinicopathologic characteristics of the study cohort

n	140 (100)
**Male sex**	80 (57.1)
**Age [years]**	62 (28 – 82)
**Disease-free interval [months]**	0 (0 – 148)
**T stage of primary**	
T1/2	18 (12.9)
T3/4	122 (87.1)
**N stage of primary**	
N0	48 (34.3)
N+	92 (65.7)
**Grading of primary**[1]	
G2	91 (79.1)
G3	24 (20.9)
**Site of primary tumor**	
Colon	96 (68.6)
Rectum	44 (31.4)
**KRAS mutation status**[2]	
Wild-type *KRAS*	47 (58.0)
Mutant *KRAS*	34 (42.0)
**Previous resection of primary**	96 (68.6)
**Multiple metastases**	83 (59.2)
Size of largest metastasis ≥ 5 cm	37 (26.4)
**Bilobar metastases**	39 (27.9)
**Extrahepatic disease**	15 (10.8)
Lungs	11 (7.9)
Peritoneum	4 (2.9)
**CEA level [µg/l]**	5.3 (0.5 – 5307.3)
**CA 19-9 level [µg/l]**	19.8 (1 – 26676.2)
**MSKCC risk score**	
0	5 (3.6)
1	25 (17.9)
2	54 (38.6)
3	38 (27.1)
4	14 (10.0)
5	4 (2.9)
**Cardiovascular comorbidity**	16 (11.4)
Coronary artery disease/Myocaridal infarction	8 (5.7)
Stroke	4 (2.9)
Deep vein thrombosis/pulmonary embolism	3 (2.1)
Peripheral artery disease	1 (0.7)
**Neoadjuvant therapy**	66 (47.1)
FOLFOX	32 (22.9)
FOLFIRI	28 (20)
FOLFOX/FOLFIRI	3 (2.1)
5-FU/Capecitabine	3 (2.1)
Bevacizumab	37 (26.4)
Cetuximab/Panintumumab	14 (10)

[1] No grading available for patients with irradiation of the primary tumor.

[2] Data missing for 59 patients.

Supplementary Table 2: Univariate analyses of CEC and CTC using various cutoff levels

	3-year survival [%]	Mean survival [months]	*P*
**CEC**			
< 12 (30th percentile)	73.8	60.4 (51.9 – 69.1)	0.03
≥ 12 (30th percentile)	52.1	48.9 (42.4 – 55.6)	
**CEC**			
< 21 (median)	73.2	60.1 (53.3 – 66.9)	0.001
≥ 21 (median)	45.2	44.7 (37.4 – 52.4)	
**CEC**			
< 35 (70th percentile)	65.6	55.1 (48.9 – 61.3)	0.02
≥ 35 (70th percentile)	39.1	44.1 (34.3 – 53.9)	
**CTC**			
< 1	64.2	53.7 (47.7 – 59.6)	0.06
≥ 1	43.9	46.1 (36.4 – 55.9)	
**CTC**			
< 2	61.4	55.2 (49.4 – 61.1)	0.03
≥ 2	40.6	39.2 (27.7 – 50.8)	
**CTC**			
< 3	60.6	54.3 (48.5 – 59.9)	0.13
≥ 3	45.4	33.4 (23.6 – 43.2)	

Data are presented as n (%) or mean (median; range); Analyses were performed using the log-rank test.
CEC, Circulating endothelial cells; CTC, Circulating tumor cells.
